# Successful use of methylene blue for severe vasoplegia in a patient with partially-treated portopulmonary hypertension with atrial septal defect undergoing liver transplantation: a case report

**DOI:** 10.1186/s12871-025-03544-7

**Published:** 2025-12-07

**Authors:** Taiga Ishihara, Gaku Kawamura, Mayuko Nagano, Seiichi Azuma, Kanji Uchida, Taro Kariya

**Affiliations:** Department of Anesthesiology and Pain Relief Center, University of Tokyo Hospital, 7-3-1 Hongo, Bunkyo-Ku, Tokyo, 113-8654 Japan

**Keywords:** Vasoplegia, Portopulmonary hypertension, Methylene blue, Liver transplantation, Case report

## Abstract

**Background:**

Vasoplegia is a life-threatening intraoperative condition. Methylene blue (MB), a potent inhibitor of nitric oxide (NO) synthase and soluble guanylyl cyclase (sGC), can treat vasoplegia but may antagonize pulmonary arterial hypertension (PAH) drugs that enhance the NO-sGC-cyclic guanosine monophosphate (cGMP) pathway. Although reports on the use of MB for vasoplegia during liver transplantation (LT) have often been published, managing vasoplegia during LT in a patient with cardiopulmonary disease, including portopulmonary hypertension (PoPH) – a subgroup of PAH – with intracardiac shunt, is challenging and has not been reported.

**Case presentation:**

We report a 57-year-old woman with PoPH, on anti-PAH selexipag with concomitant atrial septal defect (ASD) undergoing deceased donor LT. After portal reperfusion, she developed refractory hypotension, in which mean arterial blood pressure (ABP) was less than 50 mmHg and the cardiac index (CI) was maintained at 5.0 L·min^−1^·m^−2^, despite high dose of vasopressin of 4 unit·h^−1^. Diagnosed with vasoplegia, she received 100 mg of MB. Mean ABP promptly improved from 45 to above 60 mmHg, systolic ABP from around 60 to above 95 mmHg, allowing immediate reduction of vasopressin to 0.5 unit·h^−1^. She was transferred to ICU with stable hemodynamics.

**Conclusion:**

MB can be lifesaving for intraoperative vasoplegia even in patients with PoPH on PAH drug therapy with an ASD, though potential drug interactions with anti-PAH drugs warrant caution.

## Background

Vasoplegia is characterized by a pathologically low systemic vascular resistance (SVR) despite normal or increased cardiac output, resulting in low blood pressure (BP) [1]_._ Surgery-related vasoplegia has been reported in cardiac surgeries and liver transplantations (LT) and is considered a downstream of ischemia–reperfusion injury [1, 2]. The nitric oxide (NO)-soluble guanylyl cyclase (sGC)-cyclic guanosine monophosphate (cGMP) axis is one of the pathways that contribute to morbid vasodilation [3]. Methylene blue (MB), an antidote for methemoglobinemia and an injectable dye for sentinel lymph node biopsy [4], induces vasoconstriction and increases BP in vasoplegic patients by potent inhibition of nitric oxide synthase (NOS) and sGC [1]. Portopulmonary hypertension (PoPH), a subgroup of group 1 pulmonary arterial hypertension (PAH) related to portal hypertension, is one of the major indications of LT [5]. Although reports on the use of MB for vasoplegia during LT have often been published, managing vasoplegia during LT in a patient with cardiopulmonary disease, including PAH and PoPH, is challenging and has not been reported. We report a successful case of deceased-donor LT (DDLT) in a patient with preoperative comorbidity of PoPH and atrial septal defect (ASD), who had been treated with a prostacyclin receptor agonist (PRA) for PAH. Severe post-reperfusion vasoplegia and hemodynamic instability were successfully managed with MB without reversing the effect of PRA or exacerbating PAH. Written informed consent for publication was obtained from the patient. This manuscript adhered to the applicable EQUATOR guidelines.

## Case presentation

A 57-year-old female with decompensated liver cirrhosis and chronic hepatitis B was scheduled to undergo DDLT. Pre-transplant evaluation with transthoracic echocardiography (TTE) 6 years before the DDLT showed secundum ASD and estimated right ventricular systolic pressure of 71 mmHg, suggesting pulmonary hypertension (PH). Right heart catheterization revealed systolic pulmonary arterial pressure (PAP) of 75 mmHg, diastolic PAP of 30 mmHg, mean PAP of 50 mmHg (≥ 25 mmHg), pulmonary artery wedge pressure of 16 mmHg, right atrial pressure of 13 mmHg, cardiac index (CI) of 3.94 L·min^−1^·m^−2^, pulmonary vascular resistance (PVR) of 320 dynes·sec·cm^−5^ (4.0 Woods units, which is ≥ 3 Woods units), and Qp/Qs of 1.77 (Qp index 3.81/Qs index 2.15 L·min^−1^·m^−2^), which confirmed the diagnosis of PAH and PoPH with concomitant ASD. The patient had undergone multidisciplinary management, including 1.2 mg of daily oral selexipag, which is a PRA. The patient improved to systolic PAP of 50 mmHg, diastolic PAP of 21 mmHg, a mean PAP of 31 mmHg, PVR of 245 dynes·sec·cm^−5^, and Qp/Qs of 1.60, a year before the DDLT. The Child–Pugh and MELD scores were 10 and 13, respectively. The patient experienced dyspnea after walking for 30 min and chest tightness after climbing more than two stories, which is considered New York Heart Association class 2. Her preoperative laboratory blood testing revealed the following: hemoglobin 12.2 g·dL^−1^, hematocrit 35.9%, platelet count 23,000 mL^−1^, serum albumin 2.9 mg·dL^−1^, creatinine 0.71 mg·dL^−1^, aspartate transaminase 37 U·L^−1^, alanine transaminase 18 U·L^−1^, sodium 140 mmol·L^−1^, potassium 3.2 mmol·L^−1^, chloride 108 mmol·L^−1^, prothrombin time-international normalized ratio 1.34, activated partial thromboplastin time 33 s, and ammonia 109 mg·dL^−1^. TTE showed a 16 × 14-mm ASD with left-to-right shunt, a left ventricular ejection fraction of 60%–70%, dilated right heart, and mild-moderate tricuspid regurgitation with estimated right ventricular systolic pressure of 46 mmHg.

In the operating room, electrocardiography, noninvasive BP, saturation of percutaneous oxygen (SpO_2_), and capnography were monitored. For induction of anesthesia, 40 mg of propofol, 0.1 mg of fentanyl citrate, and 70 mg of rocuronium bromide were administered. The patient was intubated using a 7-mm inner diameter endotracheal tube. Anesthesia maintenance was achieved with sevoflurane at 1.2%, and boluses of fentanyl were administered up to 0.9 mg. An 18-gauge intravenous line, large-bore venous sheath, and 22-gauge arterial line were placed. Central venous pressure (CVP) was monitored via the right internal jugular vein. A Swan-Ganz catheter was placed in the right pulmonary artery. Mixed venous oxygen saturation and the thermodilutional CI were continuously monitored. Transesophageal echocardiography (TEE) was not performed. At our institution, TEE is generally avoided during LT due to the risk of pharyngoesophageal bleeding. In this case, we decided not to use TEE based on the following: PH could be assessed via Swan-Ganz; the ASD was not large, and potential significant reversal shunt would be detectable by a drop in SpO₂. Preoperative TTE showed no major RV dysfunction, and intraoperative management was considered feasible using the relationship between PAP and CVP under the supervision of an accompanying cardiac anesthesiologist. Low-dose dobutamine (3㎍·kg^−1^·min^−1^), phenylephrine (up to 0.5 mg·h^−1^), and vasopressin (0.3 unit·h^−1^) were started to maintain mean arterial BP (ABP) and RV-pulmonary artery coupling. The explantation of the recipient's liver proceeded as planned. The hepatic vein, portal vein, hepatic artery, and bile duct were anastomosed.

**Fig. 1:**
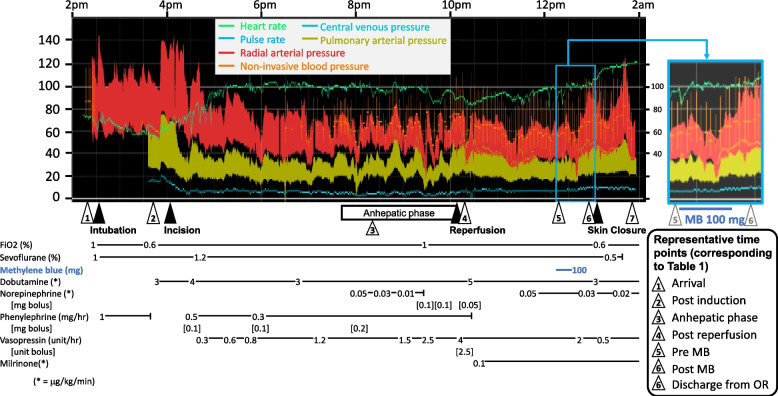
Intraoperative vital signs and administration of major drugs. Major drugs and their doses are shown in the lower panel. After portal reperfusion ("Reperfusion"), systemic hypotension persisted despite vasopressin (4 unit·h^−1^) and norepinephrine (0.5 μg·kg^−1^·min^−1^) infusion. Transfusion amount was adequate. Methylene blue (100 mg) was administered, and the systolic arterial blood pressure was restored to the 100 s and the mean arterial blood pressure to the 60 s (in the blue rectangle and magnified in the right panel). Pressors were able to be reduced immediately thereafter. White triangles are representative time points whose numbers correspond to Table 1. Detailed hemodynamic indices of these time points are shown in Table 1. FiO2 = fraction of inspired oxygen, MB = methylene blue. *calculated mixed vein

Because continuous norepinephrine (NE) administration exacerbated PH, vasopressin became the mainstay for maintaining ABP, and low-dose dobutamine was added from the anhepatic phase. After the portal reperfusion, mean ABP ranged from 40 to 51 mmHg, systolic ABP 60’s, CVP 7–9, which derived a low SVR of 384–544 dynes·sec·cm^−5^, along with high CI of 4.9–7.0 L·min^−1^·m^−2^ and assumptive Qp/Qs of 1.6, despite high-dose vasopressin (4 unit·h^−1^) and adequate transfusion. The patient was diagnosed with severe vasoplegia, and 100 mg of MB was administered intravenously over 30 min. Since MB had not previously been used for any intraoperative vasoplegia cases at our institution, continuous infusion was chosen instead of bolus, so that the drug could be discontinued immediately if any adverse reaction occurred. The inhaled nitric oxide (iNO) device was prepared in parallel in case of non-responsiveness to methylene blue. We ultimately did not use iNO, as ABP control was prioritized and adequately maintained with MB. The systolic ABP was restored to the 100 s and the mean ABP to the 60 s immediately after the completion of MB administration. Pressors were reduced immediately thereafter, and the patient was transferred to the postoperative intensive care unit (ICU) with 0.5 unit·h^−1^ of vasopressin and 0.03 μg·kg^−1^·min^−1^ of NE as pressors. SpO_2_ remained above 92% in post-reperfusion phase. Figure 1 shows intraoperative trends in vital signs and major drug administration, and Table 1 summarizes hemodynamic indices and vasoactive agents at representative intraoperative time points.

**Table 1:** Detailed hemodynamic indices and vasoactive agents at representative time points in the surgery

	**Preoperative without PRA**	**Preoperative with PRA**	**Arrival (1)**	**Post induction (2)**	**Anhepatic phase (3)**	**Post reperfusion (4)**	**Pre MB (5)**	**Post MB (6)**	**Discharge from OR (7)**
ABP (mmHg)	115/66/82**	106/70/82**	123/59/83	109/61/78	70/46/55	42/30/36	68/38/50	104/47/63	94/47/61
CI (L·min^−1^·m^−2^)	3.9	2.7		2.6	3.3	6.0	5.0	6.4	
PAP (mmHg)	75/30/50	50/21/31		65/29/43	34/20/27	45/21/31	33/19/25	50/23/32	45/24/31
SvO_2_ (%)	82	76		89	89	93	88	86	
Pressor/inotropic support									
Phenylephrine (mg·h^−1^)	₋	₋	₋	1	0.3	₋	₋	₋	₋
Dobutamine (*)	₋	₋	₋	₋	3	5	5	3	3
Vasopressin (unit·h^−1^)	₋	₋	₋	₋	1.2	4	4	0.5	0.5
Norepinephrine (*)	₋	₋	₋	₋	₋	₋	0.05	0.03	0.02
Milrinone (*)	₋	₋	₋	₋	₋	₋	0.1	0.1	0.1

Hemodynamic indices and amount of vasoactive agents at each representative time points are shown. Pressors were able to be reduced immediately after the administration of methylene blue. The numbers in parentheses at each time point correspond to those shown in Fig. 1. Pressures are expressed as [systolic/diastolic/mean] fashion

*PRA* Prostacyclin receptor agonist, *ABP* Arterial blood pressure, *MB* Methylene blue, *OR* Operation room, *PAP* Pulmonary arterial pressure, *CI* Cardiac index from continuous thermodilution method, *SvO*_*2*_ mixed venous oxygen saturation

^*^μg/kg/min

^**^non-invasive blood pressure

The total anesthesia time was 11 h and 39 min. Blood loss was 34,100 mL. Blood transfusions given were 7,000 mL of red blood cells, 7,680 mL of fresh frozen plasma, 1,000 mL of platelet concentrate, and 3,600 mL of cell-salvaged blood along with 7,000 mL of 5% albumin as colloid. The patient was transferred to the ICU with intubation as planned. Sufficient portal flow (40–50 cm·sec^−1^) was measured throughout the neo-hepatic phase and the ICU stay using Doppler ultrasonography.

The patient was extubated on postoperative day (POD) 5 and was transferred to the general ward on POD 15. Persistent ascites and pleural effusion necessitate a longer postoperative hospital stay. The patient was discharged home on POD 138.

## Discussion and conclusions

**Fig. 2:**
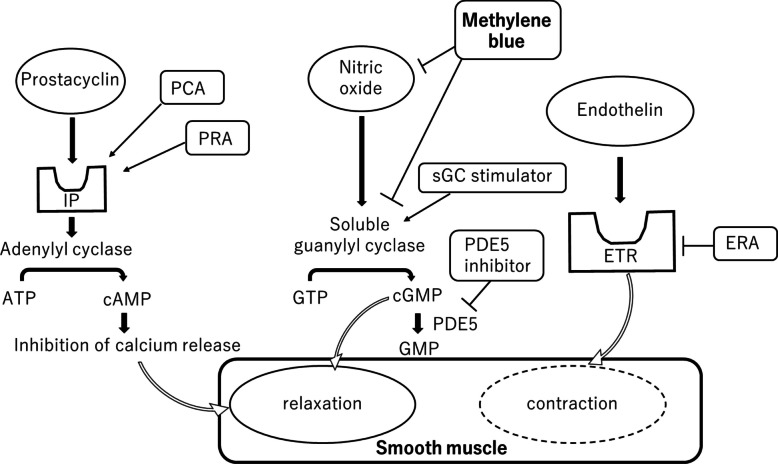
The targets of pulmonary arterial hypertension drugs and methylene blue. Pulmonary arterial hypertension drugs target endothelin, NO-sGC-cGMP, and prostacyclin pathways, enhancing smooth muscle relaxation or inhibiting smooth muscle contraction. Methylene blue is a potent NOS-sGC inhibitor and may exacerbate pulmonary hypertension in patients treated with anti-PAH NO-sGC-cGMP enhancers. ATP = adenosine triphosphate, cAMP = cyclic adenosine monophosphate, cGMP = cyclic guanosine monophosphate, ERA = endothelin receptor antagonist, ETR = endothelin receptor, GMP = guanosine monophosphate, GTP = guanosine triphosphate, IP = prostacyclin receptor, PCA = prostacyclin analogue, PDE5 = phosphodiesterase type 5, PRA = prostacyclin receptor agonist, sGC = soluble guanylate cyclase

PAH is defined as a condition in which the mean PAP is more than 20 mmHg at rest, which is caused by increased PVR and a left ventricular end-diastolic pressure of less than 15 mmHg [6]. In PAH, excessive pulmonary vasoconstriction and increased PVR lead to progressive pulmonary vascular remodeling [6, 7], resulting in right-sided heart failure [6, 7]. PoPH is classified as a subgroup of group 1 PAH if concomitant portal hypertension is present. Elucidation of these PAH pathophysiologies led to modern therapeutic approaches targeting the endothelin, NO-sGC-cGMP, and prostacyclin (prostaglandin I_2_, PGI_2_) pathways (Fig. 2) [6, 8]. Drugs targeting these three pathways are currently clinically available: endothelin receptor antagonist (ERA) for the endothelin pathway, phosphodiesterase 5 inhibitor and sGC stimulator for the NO-sGC-cGMP axis, and prostacyclin analog (PCA) and PRA for the PGI_2_ pathway. Savale et al. reported that the survival probability in patients with PoPH was closely linked to the severity of liver disease, with those undergoing LT achieving significantly better long-term outcomes than those who did not [9]. Favorable short-term outcome of LT was reported in patients with PoPH with mean PAP < 35 mmHg and PVR < 400 dynes·sec·cm^−5^. LT is contraindicated in patients with a mean PAP > 50 mmHg [5]. Mortality depends on the PVR and right-sided heart function. Preoperative PRA lowered the patient’s mean PAP to less than 31 (< 35) mmHg with decrease of PVR to 245 (< 400) dynes·sec·cm^−5^ as described in Case Presentation. PoPH with high PVR had been improved with preoperative PRA, which enabled the patient to undergo LT.

Vasoplegia was diagnosed intraoperatively. Patarroyo et al. proposed a clinically relevant definition of vasoplegia following cardiac surgery [10] in combination with the most cited definitions. The proposed definition was as follows – persistent low SVR (< 800 dynes·sec·cm^−5^) despite multiple intravenous pressor drugs at high dose (epinephrine ≥ 4 μg·min^−1^, NE ≥ 4 μg·min^−1^, dopamine ≥ 4 μg·min^−1^, vasopressin ≥ 1 unit·h^−1^) with preserved CI (> 2.5 L·min^−1^·m^−2^). If this definition can be extended to non-cardiac cases, our case fits these criteria. The patient’s intraoperative arterial blood pH showed around 7.25 throughout the procedure, and bladder temperature was 36.6–36.8℃. These observations suggest that while catecholamines were pharmacologically active, they failed to adequately overcome vasoplegia. Ischemia–reperfusion injury might play a significant role as the cause of vasoplegia.

MB can be a magic bullet for vasoplegia [1] but should theoretically be used with caution if the patient is on PAH drugs. Since MB is a potent NOS-sGC inhibitor, MB therapy may exacerbate PH in patients treated with anti-PAH NO-sGC-cGMP enhancers. To the best of our knowledge, this is the first report on the use of MB in a patient with PAH and systemic vasoplegia. There has been one communication on the theoretical concern of MB in worsening PH in severe PH patients [11, 12] and a description of the possibility of a PVR increase [3]. Our patient was on selexipag, a PRA in which the anti-PAH effect was not targeted by MB. Preoperative PGI_2_ therapy may control the intraoperative PH and result in an acceptable ABP/PAP ratio after MB. In our patient, MB increased SVR by 12% and estimated stressed blood volume [13] by 25%. Moreover, the patient had an ASD, which caused a left-to-right shunt and compromised hemodynamics. Hemodynamic computer simulation using Harvi [14] indicated that these changes in BP and resistance affect Qp/Qs less than 5%, which is compatible with no intraoperative profound SpO_2_ decline.

Dobutamine and low-dose milrinone were used to support RV contractility because RV dilatation strongly suggests chronic overload, remodeling, impaired pump reserve, and vulnerability. Given the potential myocardial depression during anesthesia, supporting RV contractility via inotropic agents and reducing RV afterload through pulmonary vasodilation using dobutamine and milrinone were considered beneficial for hemodynamic management.

In conclusion, the combination of MB and vasopressors successfully saved severe post-reperfusion vasoplegia in a patient with DDLT with preoperative PoPH and ASD without exacerbating PAH. If a patient with PAH undergoes surgery at risk of vasoplegia, including LT, the class of PAH drugs administered should be recognized in preoperative evaluation to avoid exacerbation of PAH during treatment of vasoplegia.

## Data Availability

Not applicable.

## Abbreviations

ABP: Arterial blood pressure
ASD: Atrial septal defect
BP: Blood pressure
CI: Cardiac index
CVP: Central venous pressure
DDLT: Deceased donor liver transplantation
ERA: Endothelin receptor antagonist
ICU: Intensive care unit
LT: Liver transplantation
MB: Methylene blue
NE: Norepinephrine
NO: Nitric oxide
NOS: Nitric oxide synthase
PAH: Pulmonary arterial hypertension
PAP: Pulmonary arterial pressure
PCA: Prostacyclin analogue
PH: Pulmonary hypertension
POD: Postoperative day
PoPH: Portopulmonary hypertension
PRA: Prostacyclin receptor agonist
PVR: Pulmonary vascular resistance
sGC: Soluble guanylyl cyclase
TEE: Transesophageal echocardiography
TTE: Transthoracic echocardiography
